# Synthesis of control unit for future biocomputer

**DOI:** 10.1186/s13036-018-0109-4

**Published:** 2018-08-14

**Authors:** Chun-Liang Lin, Ting-Yu Kuo, Wei-Xian Li

**Affiliations:** Department of Electrical Engineering, National Chung Hsing University, Taichung, 402 Taiwan

**Keywords:** Synthetic biology, Genetic logic circuit, Arithmetic and logical unit, Control unit

## Abstract

**Background:**

Synthesis of a variety of biological circuits for specific functional purposes has made a tremendous progress in recent years. The ultimate goal of combining molecular biology and engineering is to realize a functional biocomputer. To address this challenge, all previous efforts work toward building up the bio-computer as the ultimate goal. To this aim, there should be a key module, named control unit (CU), to direct a serious of logic or arithmetic operations within the processor.

**Methods:**

This research task develops a bio-CU to work with a bio-ALU, which is realized from the combination of previously developed genetic logic gates to fulfill the kernel function of CPU as those done in the silicon computer.

**Results:**

A possible framework of the bio-CPU has demonstrated how to connect a bio-CU with a bio-ALU to conduct a fetch-decode-execute cycle of a macro instruction. It presents not only capability of 4-bit full adder but coordination of related modules in biocomputer.

**Conclusions:**

We have demonstrated computer simulation for applications of the genetic circuits in biocomputer construction. It’s expected to inspire follow-up study to synthesize potential configurations of the future biocomputer.

## Background

Synthesis of a variety of biological circuits for specific functional purposes has made a tremendous progress in recent years. The ultimate goal of combining molecular biology and engineering is to realize the biocomputer [1–3]. In particular, synthesis of fundamental Boolean logic gates [4] and design of genetic oscillator [5, 6] have been successfully or partly successfully reached since the earliest research effort focused on creating oscillating behavior of a genetic circuit in 2000 by Elowitz and Leibler [7]. Basically, a biological circuit is different from its counterpart in the digital logic circuit; the former uses chemical reaction of gene expression to simulate on and off states of the protein concentration [8] with DNA-binding proteins and DNA-binding factors expressing protein concentration for a specific logical function [9]. With the rapid development of synthetic biology, a class of combinational and sequential genetic logic circuits [10–12] have been developed toward specific applications. Fast grow of synthetic biology is because most of the biochemical reactions can be described in terms of mathematical models through the use of non-linear Hill differential equations [13] and computer simulation before conducting real-world experiments.

All of the efforts work toward building up a functional bio-computer as the ultimate goal [14, 15]. To this aim, there should be a key module, named control unit (CU), to direct a serious of logic or arithmetic operations within the processor. It guides the computer’s memory, arithmetic/logic unit (ALU) and input/output devices to respond macro instructions by arranging timing and control signals.

Following our previous works [16–21], we continue to develop a bio-CU working with the bio-ALU, which is realized from the combination of previously developed genetic logic gates to fulfill the kernel function of CPU as in the silicon computer. A standard CPU consists of three cores: CU, ALU and memory. The ALU is triggered by the CU when it receives instructions. An instruction cycle including “fetch-decode-execute” is the standard operating procedure of the CPU. That is, fetching data from memory, decoding instruction into executable commands and executing the instruction by the ALU. The result is next stored in a temporary register for further processing. Once the process is completed, the CPU sets itself up to wait for the next instruction to come.

**Fig. 1:**
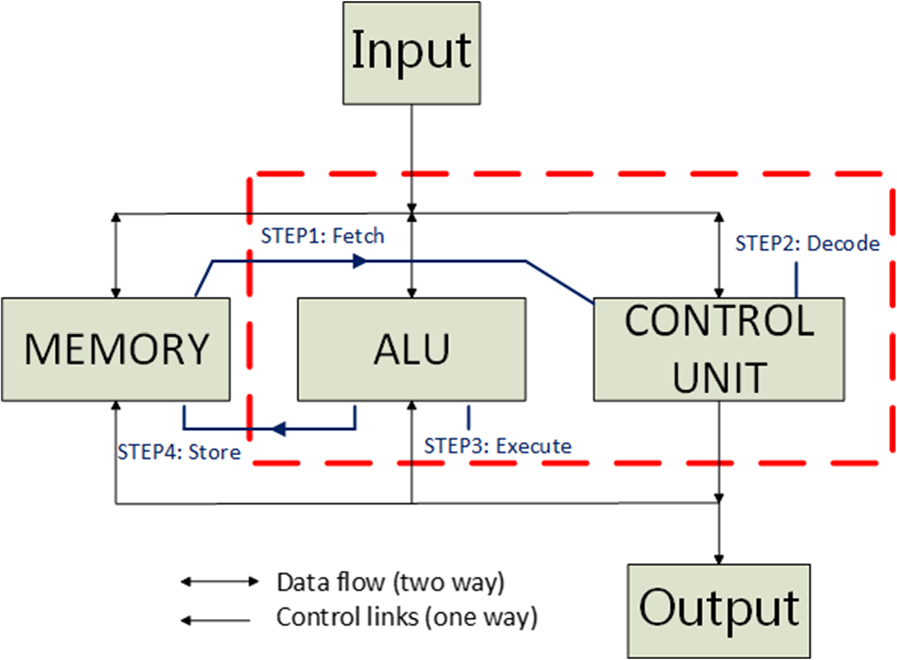
Instruction flow inside the biocomputer. It shows the instruction flow that operates in the biocomputer, the process of the Decode and Execute are operated in the ALU and CU

In this research task, we propose a possible framework of the bio-CPU to demonstrate how to connect a bio-CU with a bio-ALU to conduct a fetch-decode-execute cycle of a macro instruction (for the demonstration purpose, shown here is only a half function of the instruction cycle), see Fig. 1. The paper presents not only the capability of 4-bit full adder but the coordination of related modules in a biological computer.

## Method

### Design of bio-register

In the silicon CPU, the inherent registers are used to temporarily store data while the computational kernel conducting logic or arithmetic operation. In general, there are instruction register, program counter, temp register, and accumulator insider a CPU. Inside of a one-bit register, there is an edge-triggered D-type flip-flop, which constitutes the fundamental unit of the register. A D-type flip-flop is made up of a NOT gate, two AND gates and four NAND gates.

#### Fundamental genetic logic gate

In the traditional electronic circuits, one uses several Boolean logic gates to realize a CPU. Before constructing a biocomputer in the genetic system, we start here by constructing a class of genetic logic gates. The genetic expression consists of the transcription and translation processes [16]. When the enzyme RNA polymerase (RNAp) is restricted to the relevant promoter, the DNA transcript is converted to messenger RNA (mRNA), and the transcription rate is controlled by transcription factors (TF). Assembling a variety of standard biological sites, including reporters of the coding regions and transcription factors, RNA, promoters, can lead to functional realization of a fundamental genetic logic gate [9].

The mathematical model describing the biochemical response of the genetic system consists of a set of two differential equations is given by [7].

1 $$ {\displaystyle \begin{array}{l}{\dot{m}}_i={\alpha}_i{f}_i(u)-{\gamma}_{m_i}{m}_i+{\alpha}_{i,0},\\ {}{\dot{p}}_i={\beta}_i{m}_i-{\gamma}_{p_i}{p}_i,i=1,\dots, L\end{array}} $$

where *m*_*i*_ and *p*_*i*_ represents, respectively, concentrations of mRNA and protein of the gene *i*, ${\gamma}_{m_i}$ and ${\gamma}_{p_i}$ represent, respectively, degradation rates of mRNA and protein, *α*_*i*_ is transcription rate of *m*_*i*_, *β*_*i*_ is synthesis rate of *p*_*i*_, *α*_*i*, 0_ is basal rate, *f*_*i*_(⋅) denotes the promoter activity function used to describe the non-linear transcriptional reactions, and *u* is concentration of TF from other inducers to control the gene expression.

**Fig. 2:**
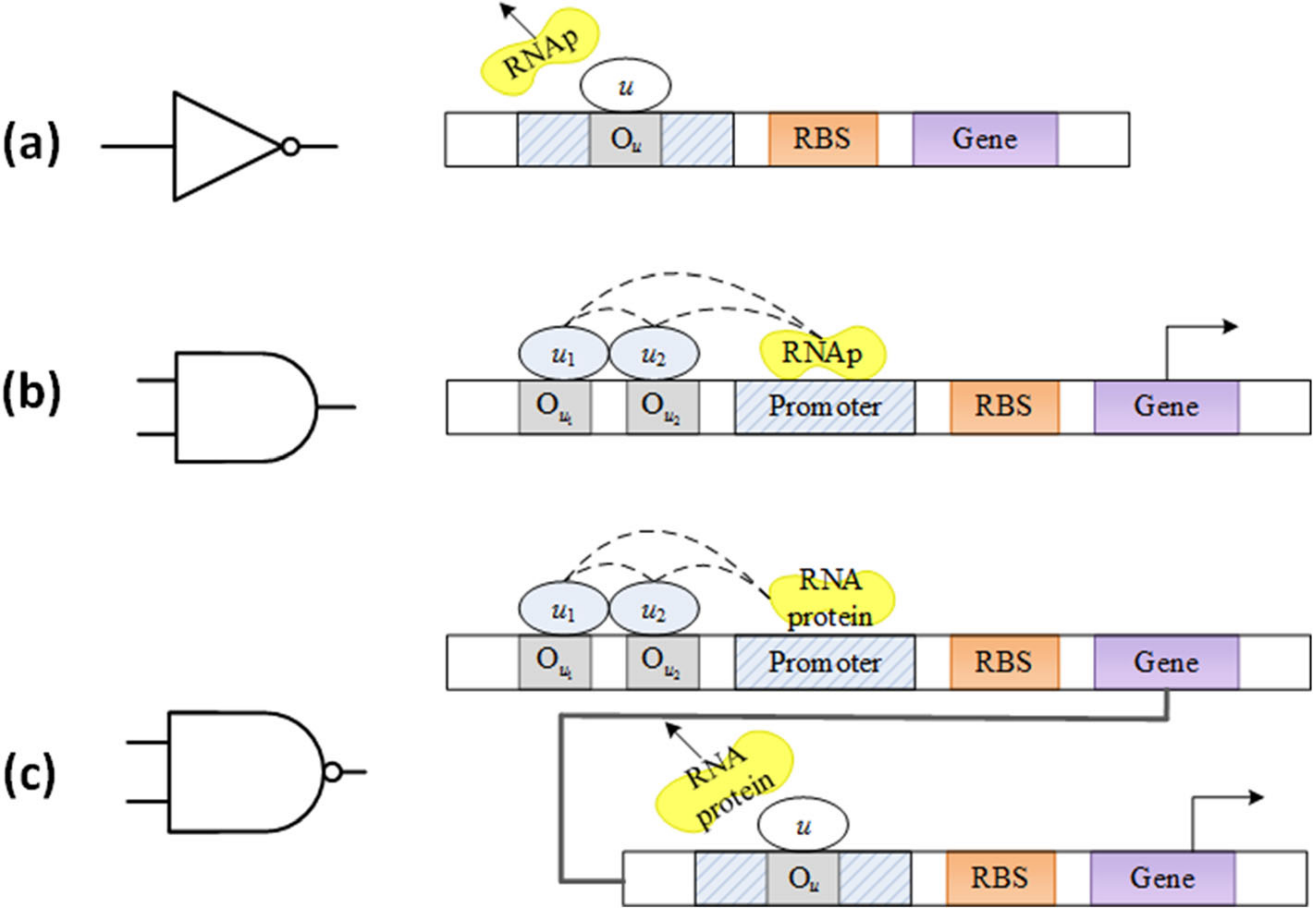
Structure of the fundamental genetic logic gates. **a** NOT gate **b** AND gate **c** NAND gate. Figure **a**, **b**, and **c** represent the genetic sequences for expressing the logic functions, respectively

A gene with an operator site can bind to a repressor or activator TF, and the promoter activity of the genetic logic NOT can be described as

2 $$ {f}_{\mathrm{NOT}}(u)=\frac{1}{1+{\left(\frac{u}{K}\right)}^n} $$

where *f*_NOT_ is a promoter activity function for logical NOT, *u* is concentration of the repressor or activator TF, *n* is Hill coefficient representing cooperatively effect between TF and the corresponding operator, and *K* is Hill constant. The binding site is inserted into the promoter region of the target gene. For the genetic expression of the logic NOT, the gene is activated by binding to the repressor TF in the corresponding operon, and its state is inactivated by binding the repressor TF. The framework is shown in Fig. 2a.

A gene with two manipulation sites can bind two repressors TFs or activator TFs, and the promoter activity of the genetic logic AND gate can be described as

3 $$ {f}_{\mathrm{AND}}\left({u}_1,{u}_2\right)=\frac{{\left(\frac{u_1}{K_1}\right)}^{n_1}{\left(\frac{u_2}{K_2}\right)}^{n_2}}{1+{\left(\frac{u_1}{K_1}\right)}^{n_1}+{\left(\frac{u_2}{K_2}\right)}^{n_2}+{\left(\frac{u_1}{K_1}\right)}^{n_1}{\left(\frac{u_2}{K_2}\right)}^{n_2}} $$

where *f*_AND_ is a logic AND promoter activity function [22], *u*_1_ and *u*_2_ are concentrations of repressor or activator TFs, *K*_1_ and *K*_2_ are Hill constants of *u*_1_ and *u*_2_, respectively, *n*_1_and *n*_2_ are the corresponding Hill coefficients. For the logic AND gate, the protein is produced only in the presence of two TFs, see Fig. 2b. The NAND gate can be implemented by cascading a NOT gate and a AND gate, see Fig. 2c.

A combination of biological registers may include at least NOT, AND, NAND, and some other fundamental logic gates. One is referred to details of the creature from [13]. For practical realization, heterogeneous regulation can be used to synthesize an AND gate in *Escherichia coli*. The AND gate includes the coactivating genes hrpR and hrpS controlled by the promoter input, and the output depends on the σ54-dependent hrpL promoter. When the ribosome binding site (RBS) serves as is used as a linear amplifier, it is applied to regulate protein expression levels. It is next to cascade a NOT gate that is assembled with the cI/Plam repressor module containing the f lambda gene cI and PR promoters.

**Fig. 3:**
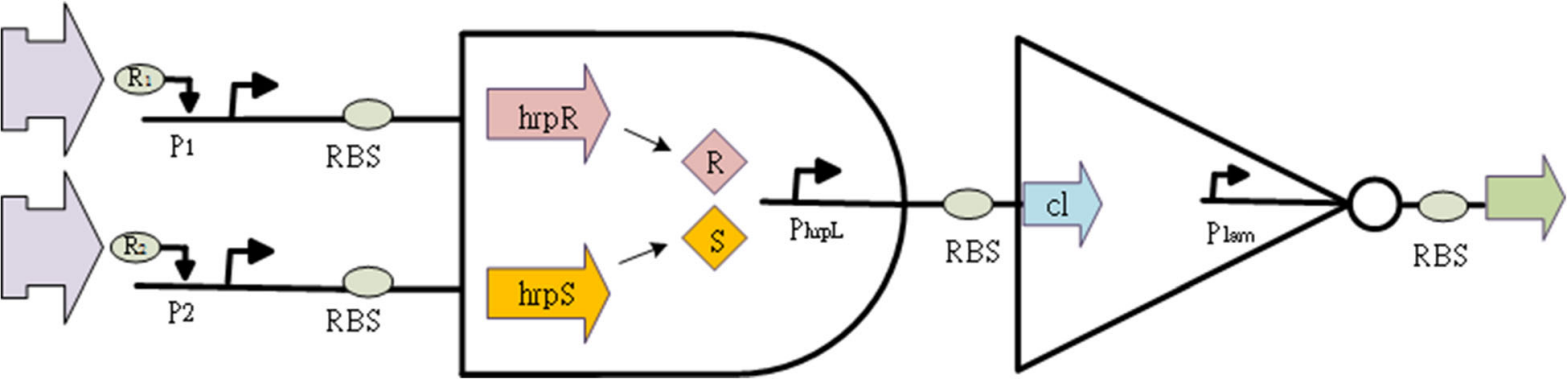
Combinational genetic NAND gate. The structure illustrates the NAND gate in the biology

By the above description, a modular combinational NAND gate can be generated as illustrated in Fig. 3. To establish the simplified model, we focus only on the steady state behavior of the mRNA as

4 $$ {m}_i=\frac{\alpha_i}{\gamma_{m_i}}{f}_i(u)+\frac{\alpha_{i,0}}{\gamma_{m_i}} $$

5 $$ {\dot{p}}_i={\alpha}_{P_i}{f}_i(u)-{\gamma}_{P_i}{p}_i+{\alpha}_{P_0,i} $$

The protein dominated response is given by the dynamic eq. (5) where the parameters $$ {\alpha}_{P_i}=\raisebox{1ex}{${\alpha}_i{\beta}_i$}\!\left/ \!\raisebox{-1ex}{${\gamma}_{m_i}$}\right. $$ and $$ {\alpha}_{P_0,i}=\raisebox{1ex}{${\alpha}_{i,0}{\beta}_i$}\!\left/ \!\raisebox{-1ex}{${\gamma}_{m_i}$}\right. $$. Established in the abbreviated dynamic equation and Fig. 3, the dynamic equation for characterizing the behavior of the genetic NAND is obtained as

6 $$ {\displaystyle \begin{array}{l}{\dot{p}}_{AND}={\alpha}_P{f}_{AND}\left({u}_1,{u}_2\right)-{\gamma}_P{p}_{NAND}+{\alpha}_{P_0, AND},\\ {}{\dot{p}}_{NOT}={\alpha}_P{f}_{NOT}\left({p}_{AND}\right)-{\gamma}_P{p}_{NOT}+{\alpha}_{P_0, NOT},\\ {}{p}_{AND}\left({u}_1,{u}_2\right)={p}_{NOT}\end{array}} $$

The correct behaviour of *p*_*AND*_(*u*_1_, *u*_2_) confirming the truth table of a NAND gate can be easily observed by considering the steady-state response of *p*_*AND*_(*u*_1_, *u*_2_) described by (6).

Before proceeding with the system design, it should first be remarked that the system architecture adopted in this research is based on assembling several fundamental logical gates and circuits we published in the previous works [10, 17, 22] without making significant changes. All of the parameters used in the gates or circuits can be found from these references.

#### Synthesis of genetic D Flip flop (D-FF)

**Fig. 4:**
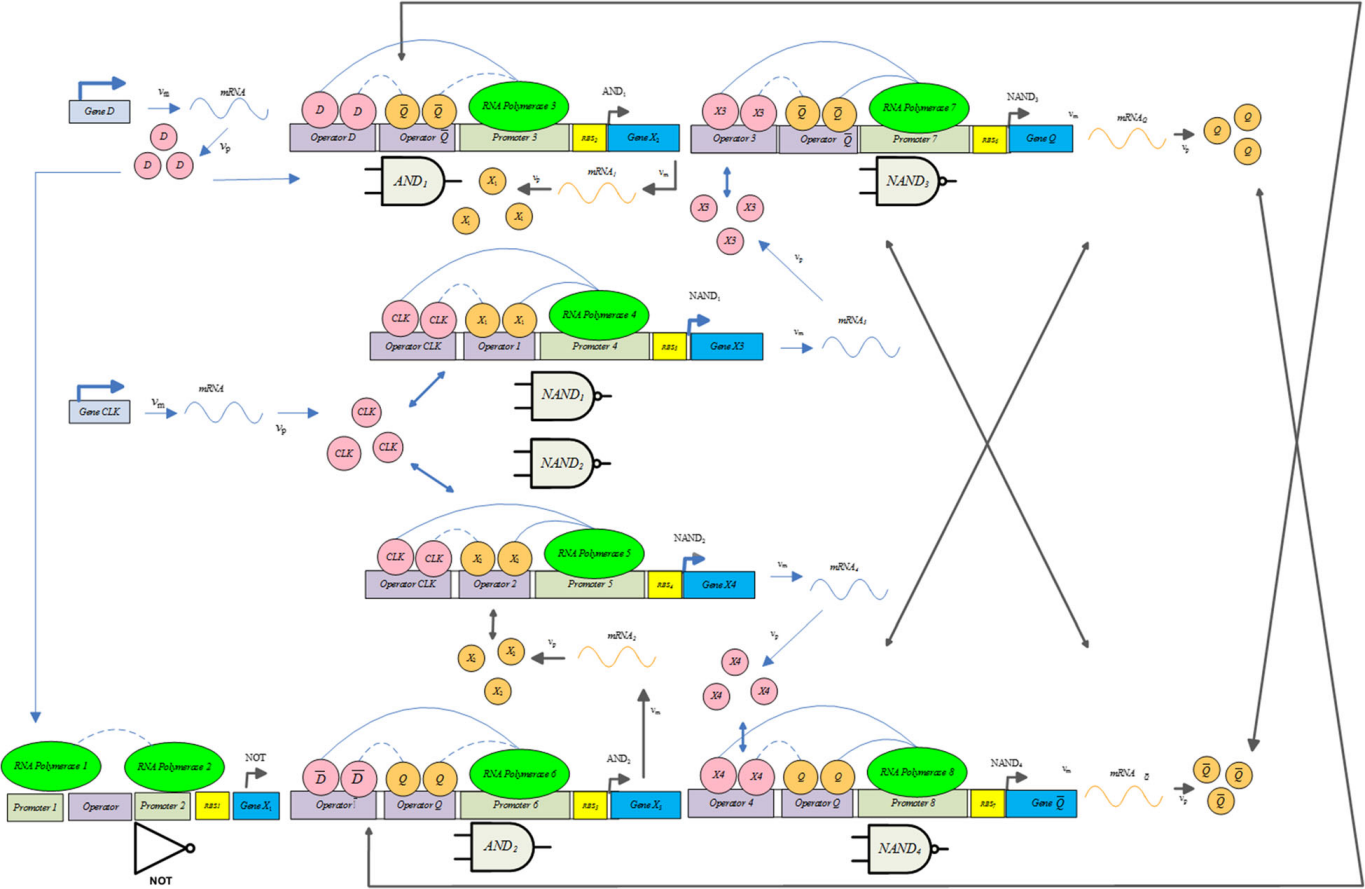
Schematic diagram of the biological D-type FF. This figure shows the D-type FF structure that is synthesized by the corresponding genetic sequences

D-FF consists of several biological logic gates with a clock input as a state triggering command. Figure 4 illustrates implementation of the equivalent biological circuit. As shown in the figure, a similar conclusion in the digital circuit can be expected by adding a high or low protein concentration as the input.

7 $$ {\displaystyle \begin{array}{c}{\dot{p}}_{AND1}={\alpha}_P{f}_{AND}\left(D,\overline{Q}\right)-{\gamma}_P{p}_{AND1}+{\alpha}_{P_0, AND1},\\ {}{\dot{p}}_{AND2}={\alpha}_P{f}_{AND}\left({p}_{NOT}(D),Q\right)-{\gamma}_P{p}_{AND2}+{\alpha}_{P_0, AND2},\\ {}Q={p}_{NAND3}\left({p}_{NAND1}\left({p}_{AND1}, clock\right),\overline{Q}\right),\\ {}\overline{Q}={p}_{NAND4}\left({p}_{NAND2}\left( clock,{p}_{AND2}\right),Q\right)\end{array}} $$

**Fig. 5:**
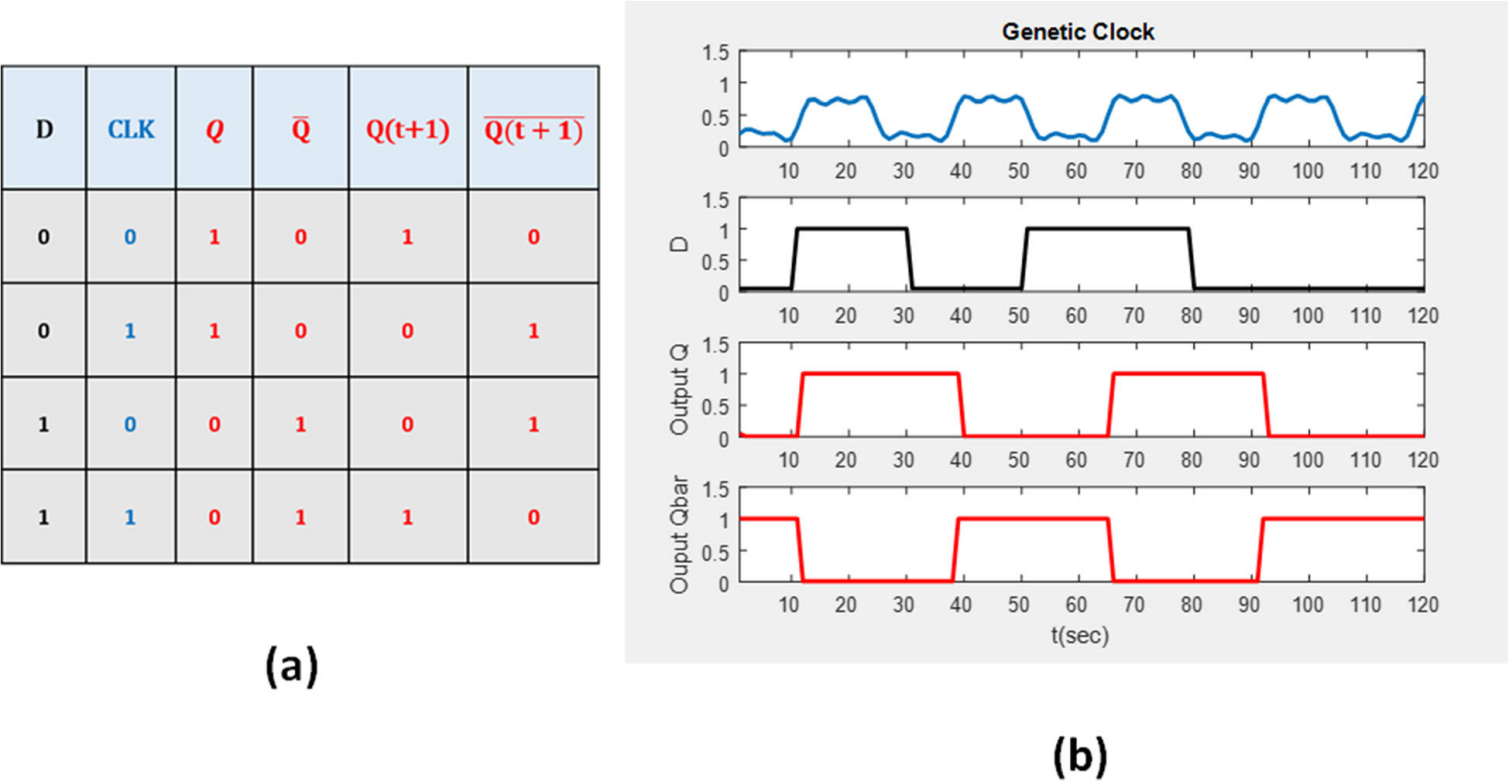
**a** Truth table of D-FF. **b** Normalized response of the biological D-FF. **a** The truth table uses 1 and 0 to represent, respectively, high and low concentration levels. **b** The output is generated by the genetic circuit that uses protein concentration to represent the state response

The edge-triggered D-FF model can be represented using the Hill differential eq. (7) where *p*_*NOT*_ is generated by ${\dot{p}}_{NOT}={\alpha}_P{f}_{NOT}\left(\cdot \right)-{\gamma}_P{p}_{NOT}+{\alpha}_{P_0, NOT}$, *D* is the protein concentration input, the clock is a periodic biological signal. Simulation results show the results of the truth table of D-FF in Fig. 5a. The normalized output response of the biological D-FF is shown in Fig. 5b. When the clock signal goes from low to high, the leading edge triggers the subsequence response where the clock signal can be generated by a genetic clock source [18, 21]. From this point of view, the stimulated concentration level changes at a time when intensity of the clock signal is enough to stimulate an accurate response. On the other hand, it is found that the signal exhibits a time delay compared to the ideal response ion the electronic circuit. This is acceptable in the biological systems because biochemical reactions are fairly slow.

### Bio arithmetic logic unit

**Fig. 6:**
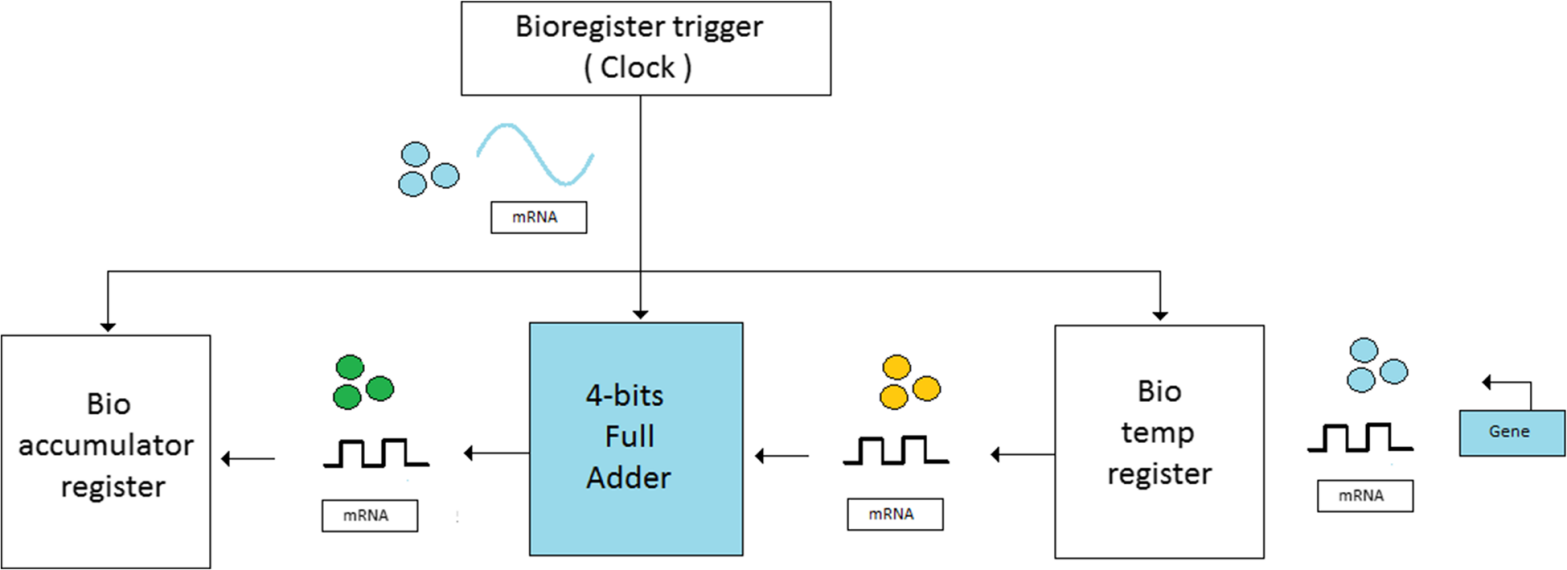
Bio-ALU schematic diagram of the operating process. It illustrates the operating flow of the bio-ALU. First, the executed data is stored to the temp register. Second, the data is propagated to the full adder for execution. Finally, the processed data is propagated to the bio-accumulator and stored in the bio-accumulator. All of the operating steps is synchronized to the clock

An ALU in the electronic circuit performs arithmetic and logical operations on the operands from the computer instruction word, such as addition or subtraction. A series of biological registers have been established in the previous work [16], and the biological arithmetic architecture has been proposed to develop basic biological computers. The simplified architecture is shown in Fig. 6. The three genetic circuits and the corresponding clock pulses are connected together. Temporary registers and genetic accumulators are created by 4-bit parallel input and parallel output (PIPO) genetic registers. In the middle is a 4-bit genetic full adder for arithmetic operations. This model is used to implement fundamental arithmetic operations in the simplest bio-computer. Here, we only focus on implementing basic full-addition arithmetic operations without considering data acquisition and storage.

#### Genetic full adder

**Fig. 7:**
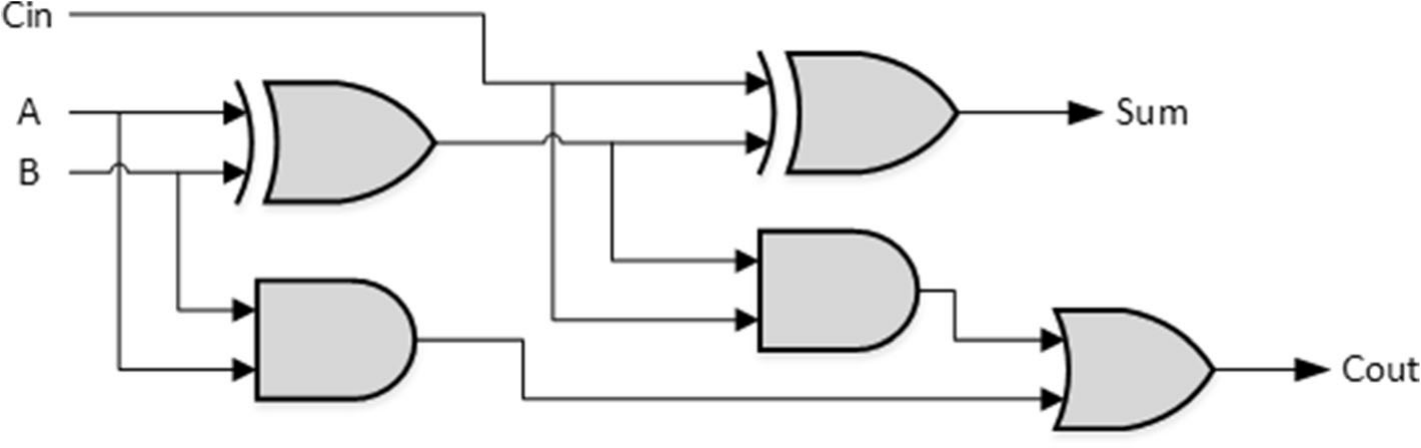
Synthesis of a full adder based on two half-adders. The structure is shown by the logic gates in electronics which is constructed by two half-adders

In the electronic circuit, mathematical addition is fulfilled by a full adder involves two half-adder in series [23]. The combination of two molecule inputs and a variety of genetic logic gates can be found from [15]. A genetic half-adder can be produced in a single mammalian cell, which produces a different reaction between erythromycin and phloretin. Through the use of natural or synthetic cell-cell communication [24], one can create a pattern formation based on functional modules. Following the previous development, we can use two gene-controlled binary adders to construct a gene binary full adder shown as in Fig. 7. As stated, a one bit genetic full-adder is fulfilled by cascading two genetic half-adders and an OR gate with the promoter activity functions for XOR gate within the half adder and the OR gate given by [25] where *f*_*OR*_ and *f*_*XOR*_ in the following equations are the promoter activity functions for logic OR and XOR, respectively. Definitions of all parameters follow those defined in (3).

8 $$ {f}_{XOR}\left({u}_1,{u}_2\right)=\frac{{\left(\frac{u_1}{K_1}\right)}^{n_1}+{\left(\frac{u_2}{K_2}\right)}^{n_2}}{1+{\left(\frac{u_1}{K_1}\right)}^{n_1}+{\left(\frac{u_2}{K_2}\right)}^{n_2}+{\left(\frac{u_1}{K_1}\right)}^{n_1}{\left(\frac{u_2}{K_2}\right)}^{n_2}} $$

9 $$ {f}_{OR}\left({u}_1,{u}_2\right)=\frac{{\left(\frac{u_1}{K_1}\right)}^{n_1}+{\left(\frac{u_2}{K_2}\right)}^{n_2}+{\left(\frac{u_1}{K_1}\right)}^{n_1}{\left(\frac{u_2}{K_2}\right)}^{n_2}}{1+{\left(\frac{u_1}{K_1}\right)}^{n_1}+{\left(\frac{u_2}{K_2}\right)}^{n_2}+{\left(\frac{u_1}{K_1}\right)}^{n_1}{\left(\frac{u_2}{K_2}\right)}^{n_2}} $$

**Fig. 8:**
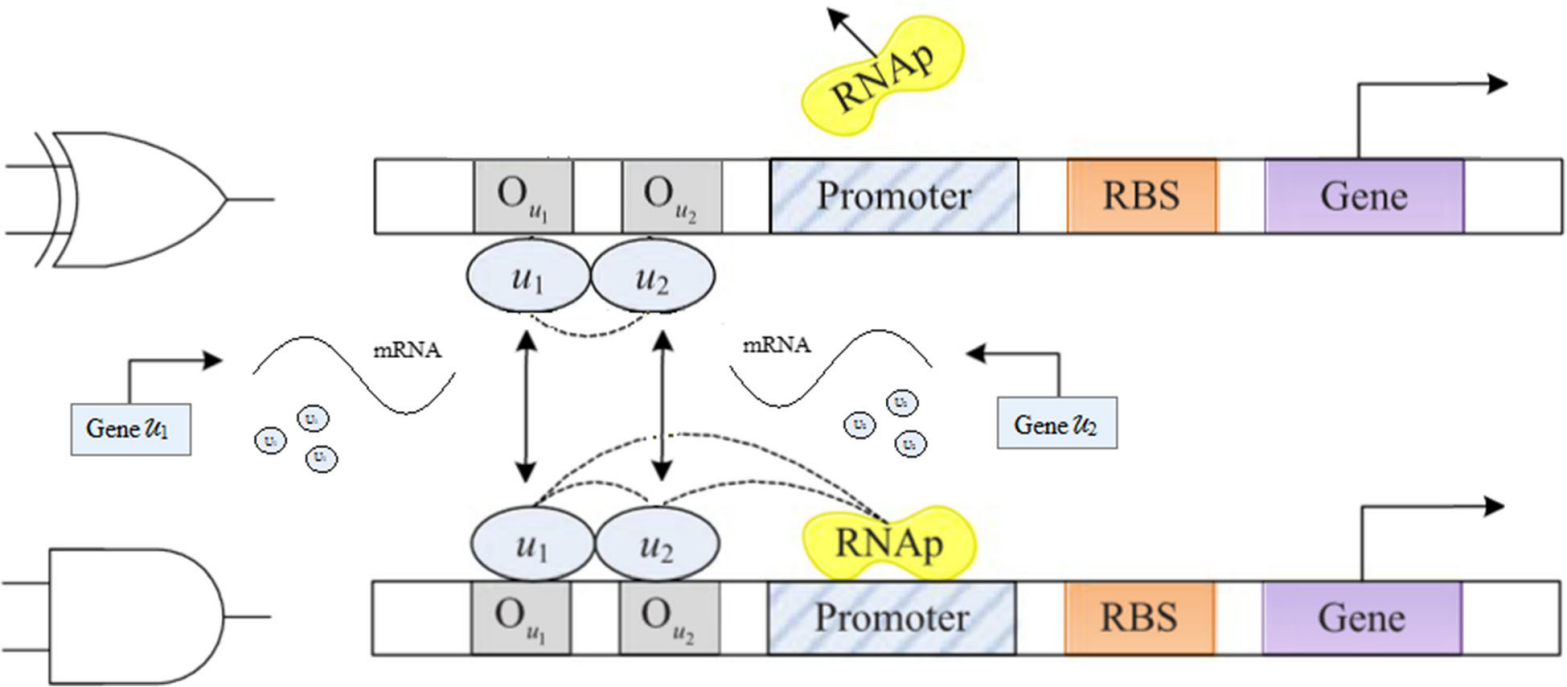
Genetic half-adder structure. The structure is constructed by the corresponding gene sequences

**Fig. 9:**
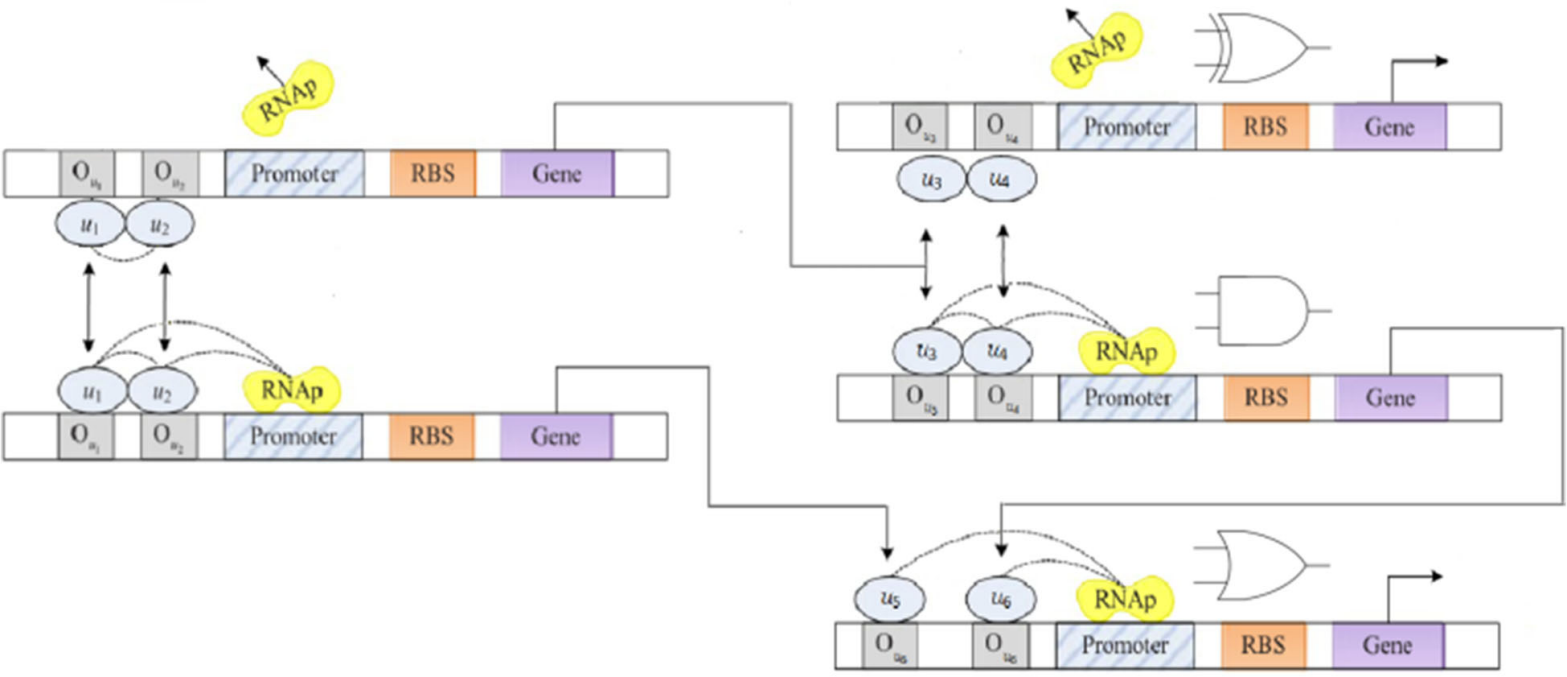
Genetic 1-bit full adder structure. The structure is constructed by the corresponding gene sequences

**Fig. 10:**
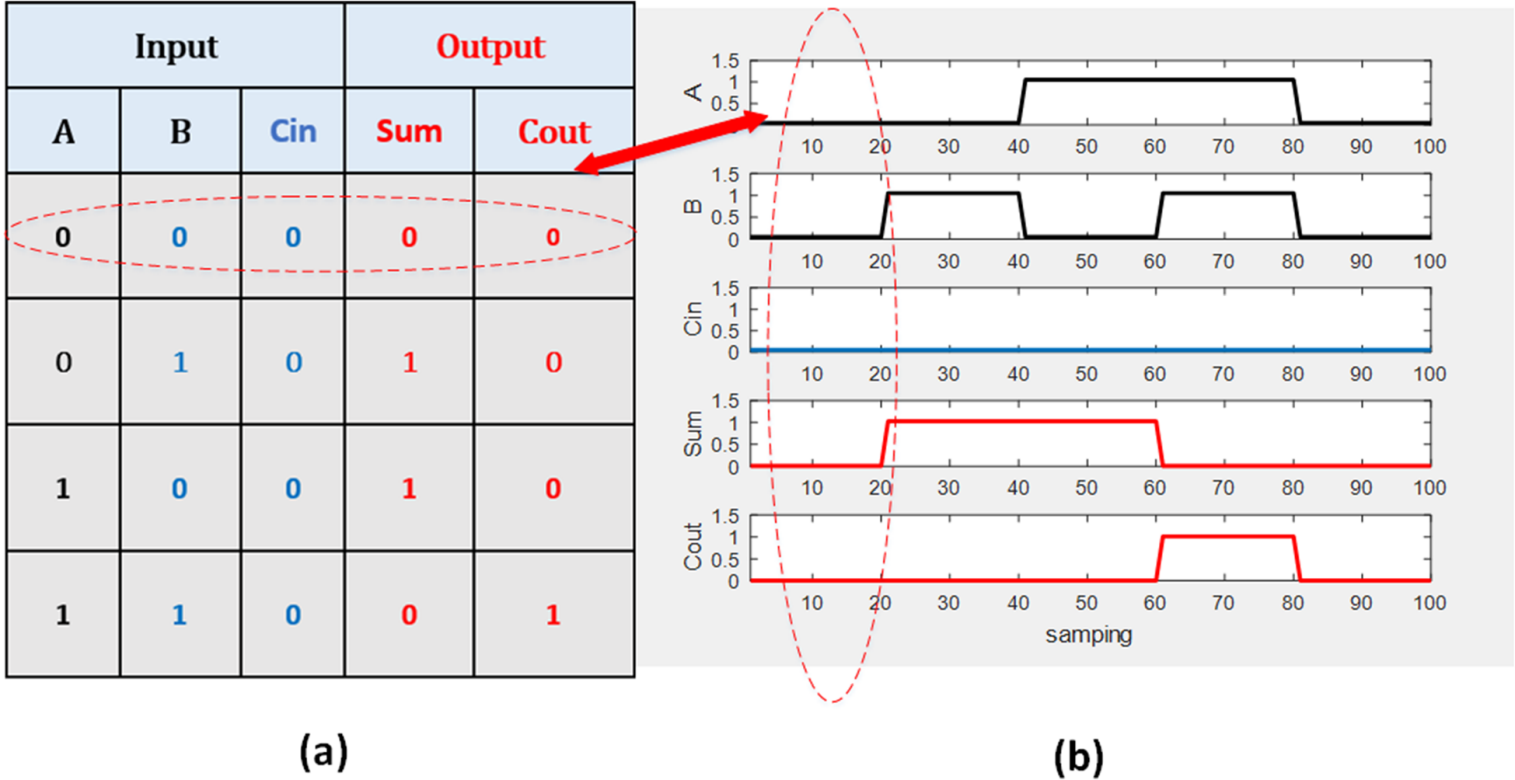
**a** Truth table of one bit full adder. **b** Genetic one bit full adder responses. **a** The table shows the result. **b** The result is produced by the genetic circuits with the dashed ellipsoid emphasizing the corresponding binary status

**Fig. 11:**
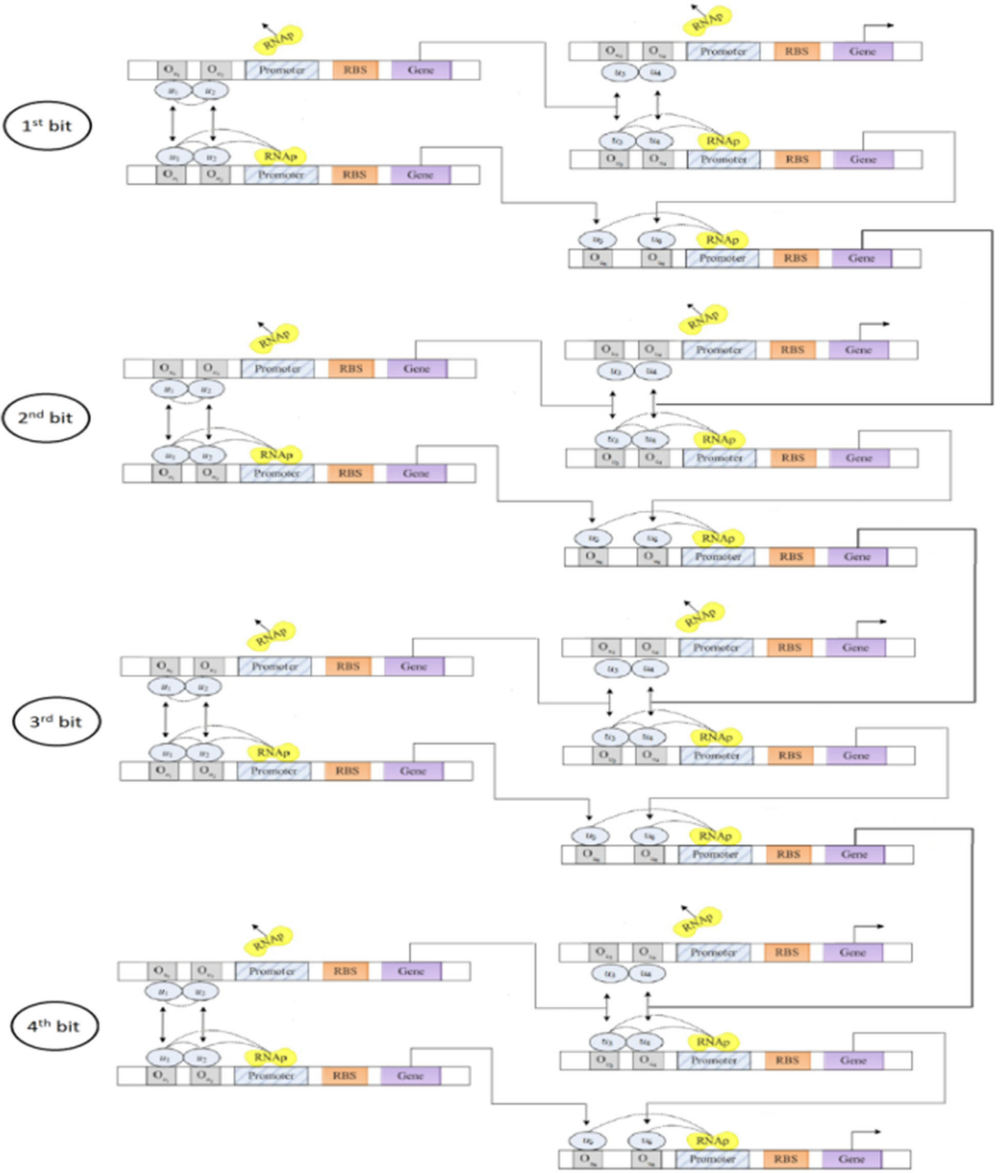
Structure of a genetic four-bit full adder. The structure is assembled by four one-bit full adders with a cascaded connection

**Fig. 12:**
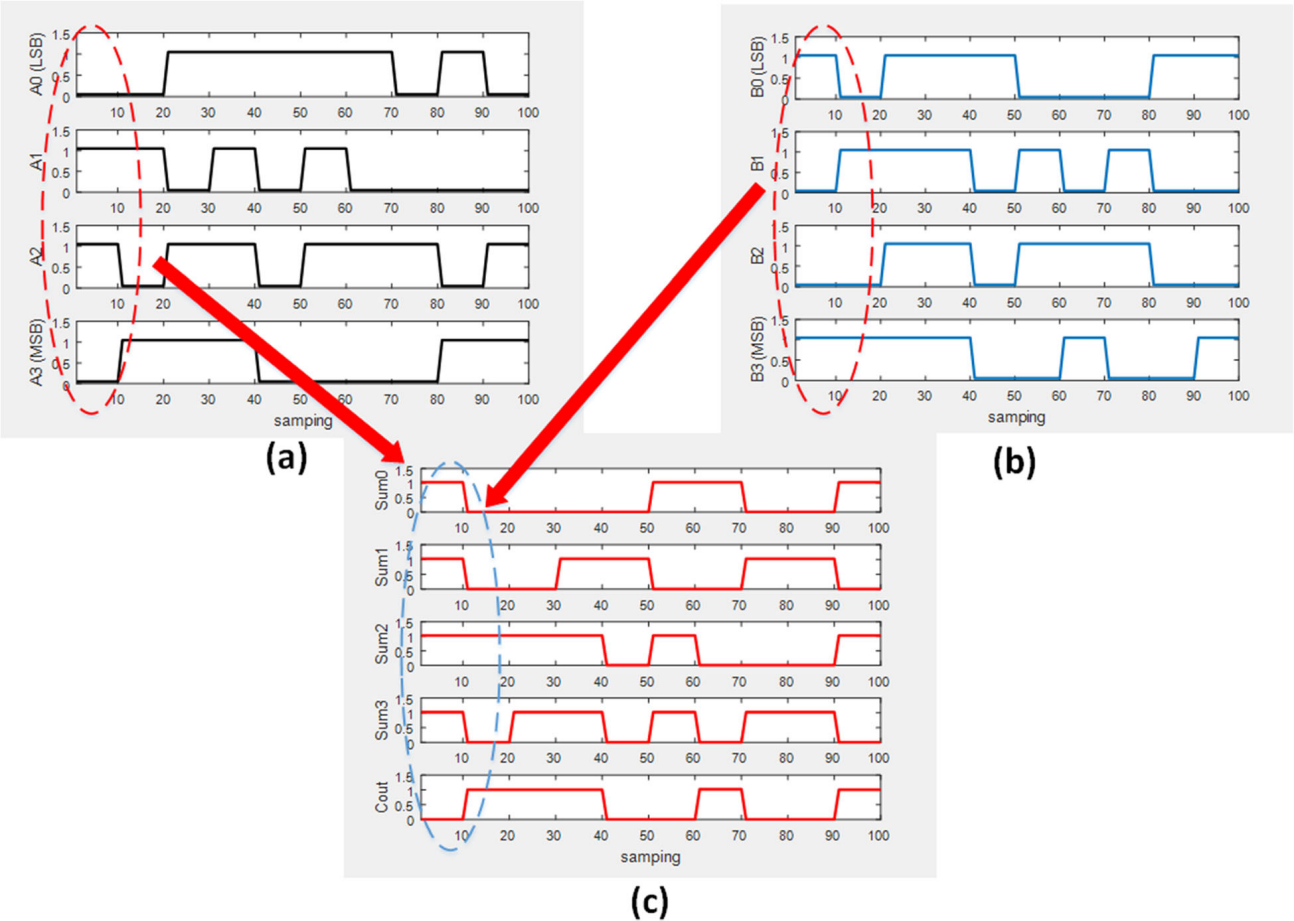
**a** Protein concentration of the four-bit augend. **b** Protein concentration of the four-bit addend. **c** Output response of the four-bit genetic full adder. The dashed ellipsoid line explains the result of addition of the augend and the addends of the first clock cycle

The output of the first half-adder will be the input to the second half-adder. Referring directly to the half-adder structure shown in Fig. 8, it can be seen that a transcription factor was used as input for transcription of the gene. A one-bit full adder is realized in Fig. 9. Based on the truth table of full adder we conduct simulation study with the results shown in Fig. 10a-b. A four-bit full adder is a direct extension of one-bit version which can be created by concatenating four one-bit genetic full adders together, see Fig. 11. Performing the simulation confirms the correct result of the system where the input augend, addend, and the output response are shown in Fig. 12a-c.

### Bio control unit

**Fig. 13:**
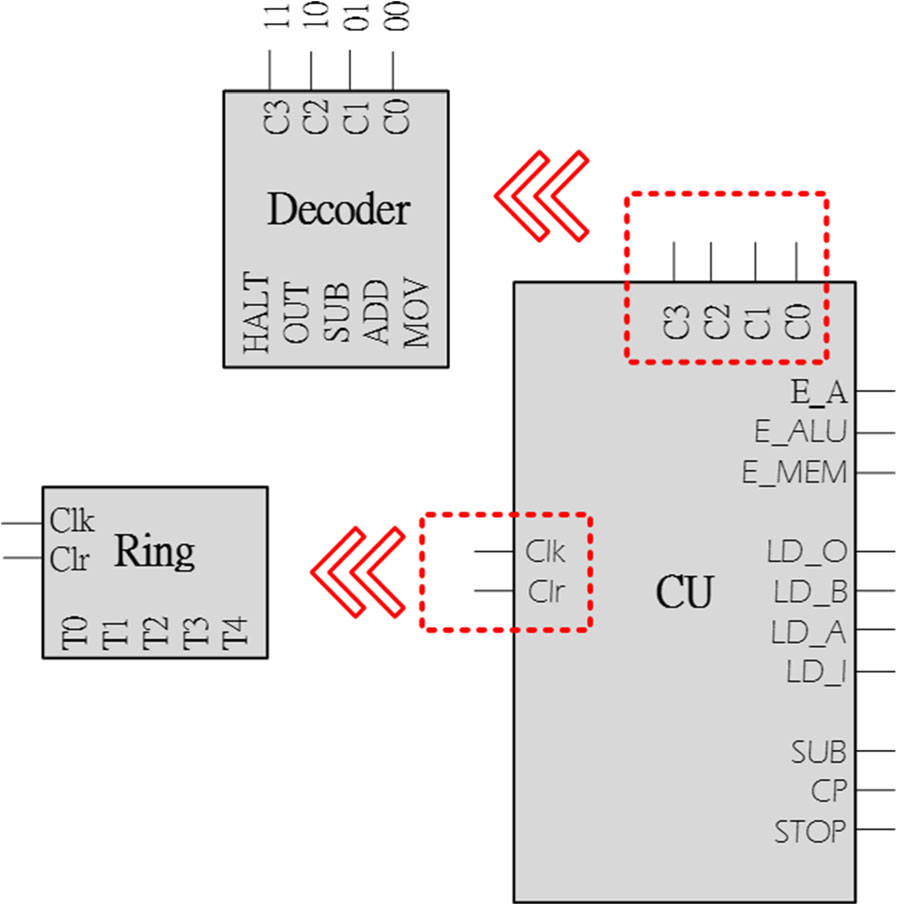
Structure of the bio-control unit. The structure works with a ring counter and the decoder

CU is a kernel of CPU that is responsible for all operations being carried out. The memory, registers and ALU will wait until CU directs the system to execute instructions. The structure of CU system can be as illustrated in Fig. 13, in which a ring counter is responsible for what instruction is the next in the operational sequence. The inputs of CU come from the instruction decoder which determine the instruction, such as MOV, ADD or SUB, to be executed. Once the instruction is received from the instruction decoder, the CU will allocate the corresponding outputs.

In the biological terms, accompanied by a genetic clock generator to generate clock signals, all of the data is originally stored in a bio-memory [26] until the bio-CU fetch and put it in a bio-temp register. Next, the data stored at the bio-temp register is moved to a genetic-full adder for execution. After the 4-bit genetic full adder accomplishes the task, the result is first moved to a genetic accumulator and next moved back to the bio-memory. The bio-CU plays the role of a commander among all functional modules. Details of all modules are described in the follows.

#### Bio ring counter

**Fig. 14:**
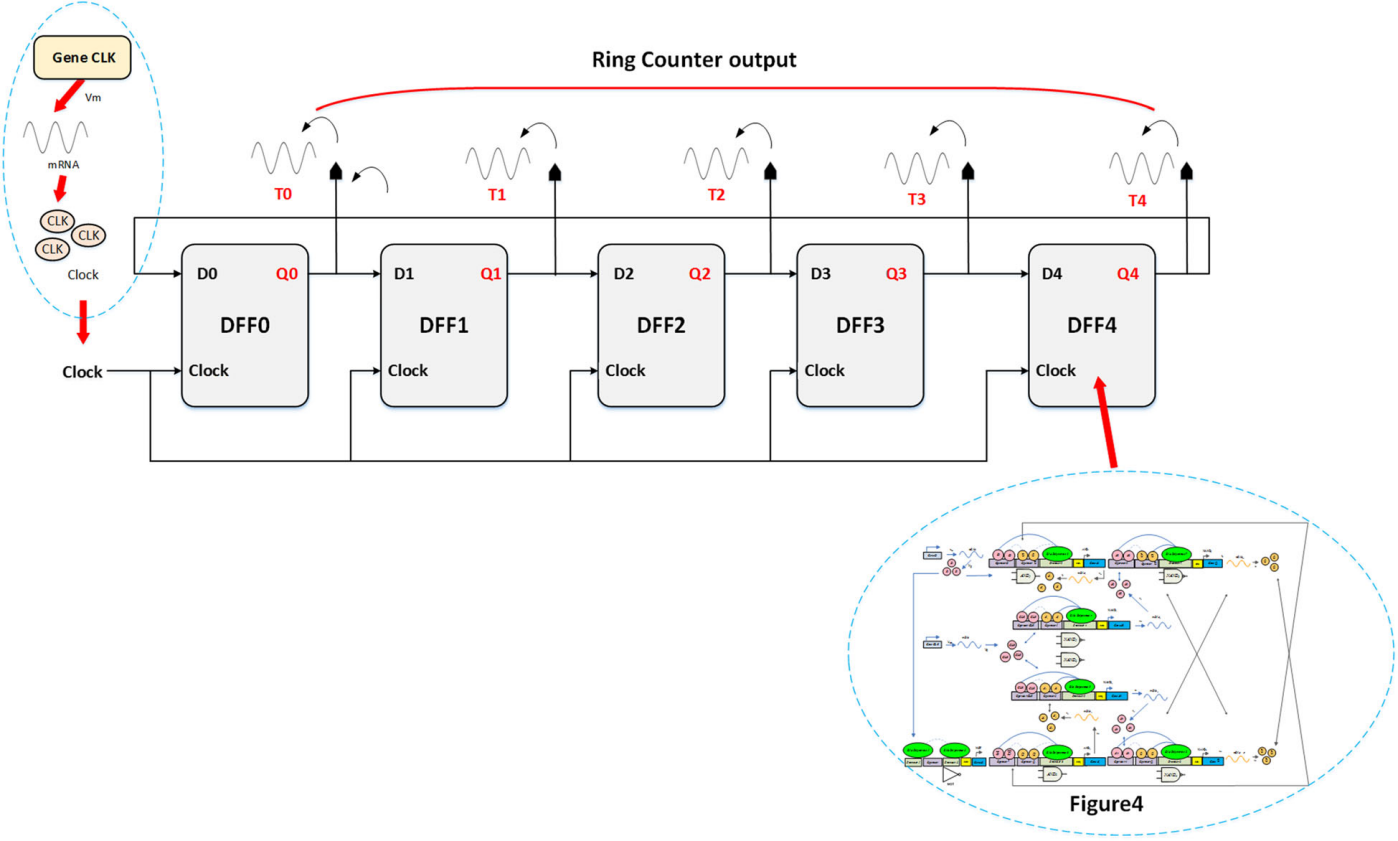
Structure of the bio-ring counter. The five-bit ring counter is cascaded by connecting five D type FFs with feedback from the last FF output to the first FF input. All FFs are triggered simultaneously by the genetic clock. It works like a shift-register with feedback

The bio-ring counter here is composed of five D-type FFs. It is an application of a shift register with the major difference being output of the last FF connected to input of the first FF, see Fig. 14. We use the ring counter as a counter to decompose the clock into four phases for instruction execution.

**Fig. 15:**
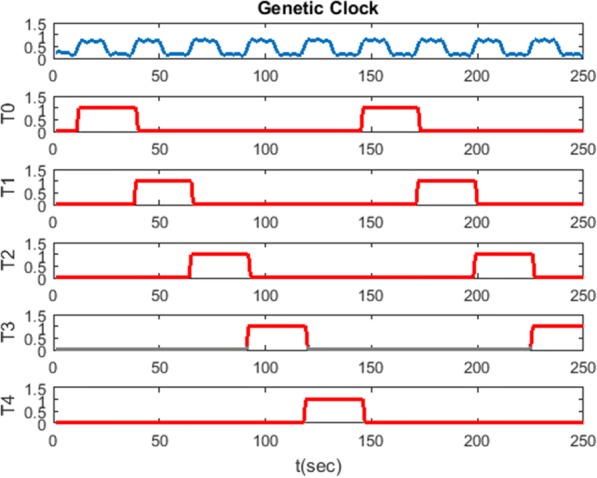
Simulation result of the bio-ring counter. The result shows the changing concentration level for each D type FF in the bio-ring counter. Each triggering state of the bio-ring counter has its specific purpose depicted in Table 2

Let’s consider the demonstration result of the ring counter shown in Fig. 15. The counter starts as the first clock comes, T0 is triggered by the first clock pulse, and T1 follows T0 while T0 goes from high to low. The process (T0 to T4) goes through 5 FFs until the last FF (T4) starts over. This five bits ring counter goes through 5 sequences within the cycle: 00001, 00010, 00100, 01000, and 10,000.

#### Bio-temporary register and bio-accumulator

The function of temporary register is to save data fetched from memory. An accumulator is a register for temporarily saving data for or after calculation from the arithmetic and logical unit (ALU). Temporary register and accumulator are also called as the operand registers because they offer operands to ALU.

We refer to Intel 4004 for construction of the basic 4-bit computer architecture. In the computer architecture, data store and fetch are important steps, so for the 4-bit register, we synthesize it by combining several genetic D-FFs, which can be realized during the biochemical reaction. The action of data read appears at the rising edge of the clock.

The reaction of the sequential process should be synchronized with the clock signal. When the trigger’s strength of the clock signal is sufficient to stimulate the exact response, the concentration level of the D-FF output follows.

**Fig. 16:**
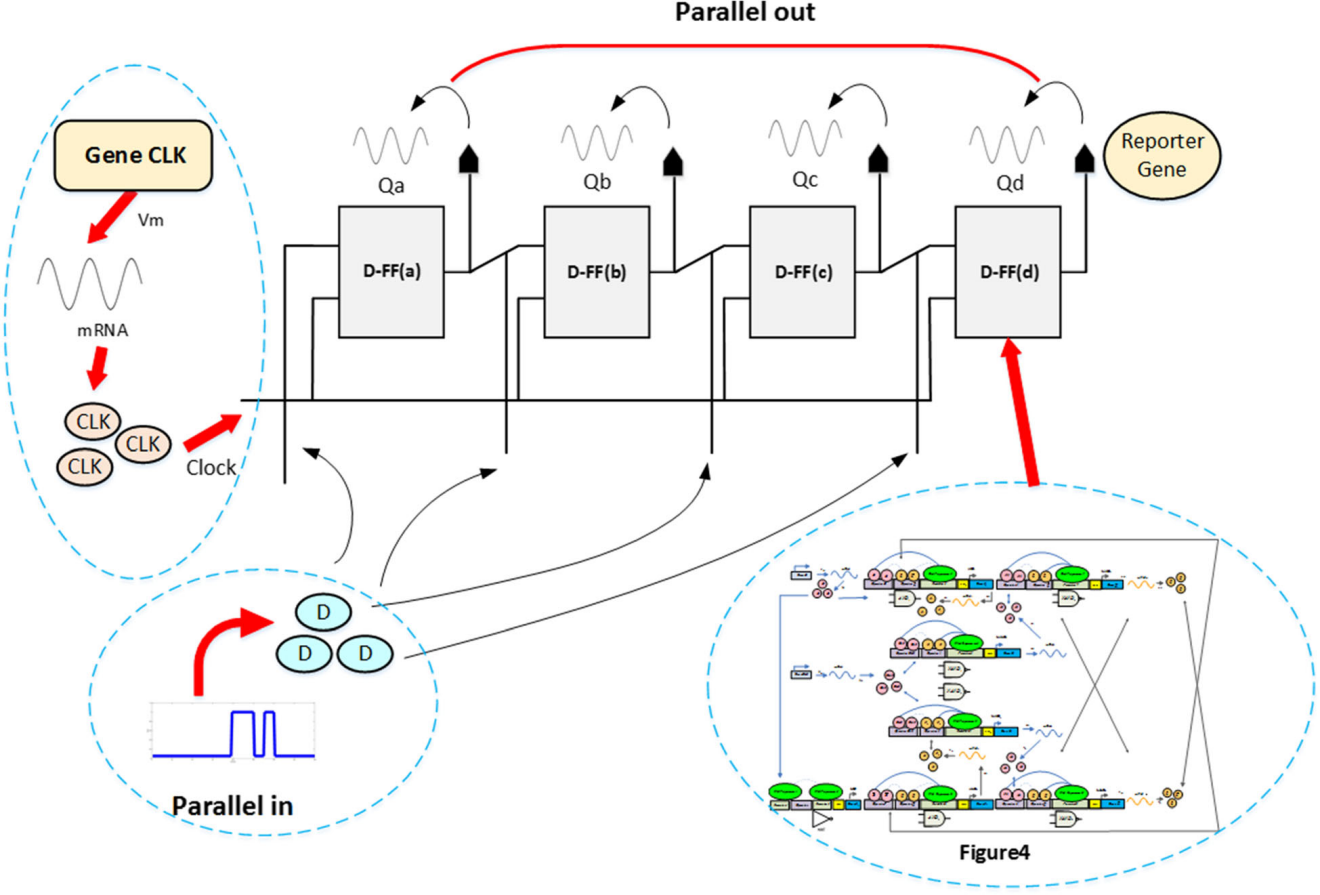
Schematic diagram of the PIPO register. The four-bit PIPO register is cascaded by connecting four D type FFs with each FF accepting data input simultaneously

For the PIPO bio-register, the 4-bit data enters in the parallel manner and is transferred in parallel to the corresponding outputs Qa to Qd in a clock pulse. Its schematic diagram is illustrated in Fig. 16.

The CU directs output of the adder to the accumulator, and data to be operated is placed in the temporary register where the 4-bit PIPO configuration is adopted to construct temporary register and accumulator which is triggered by a clock signal generator to synchronize state transmission.

**Fig. 17:**
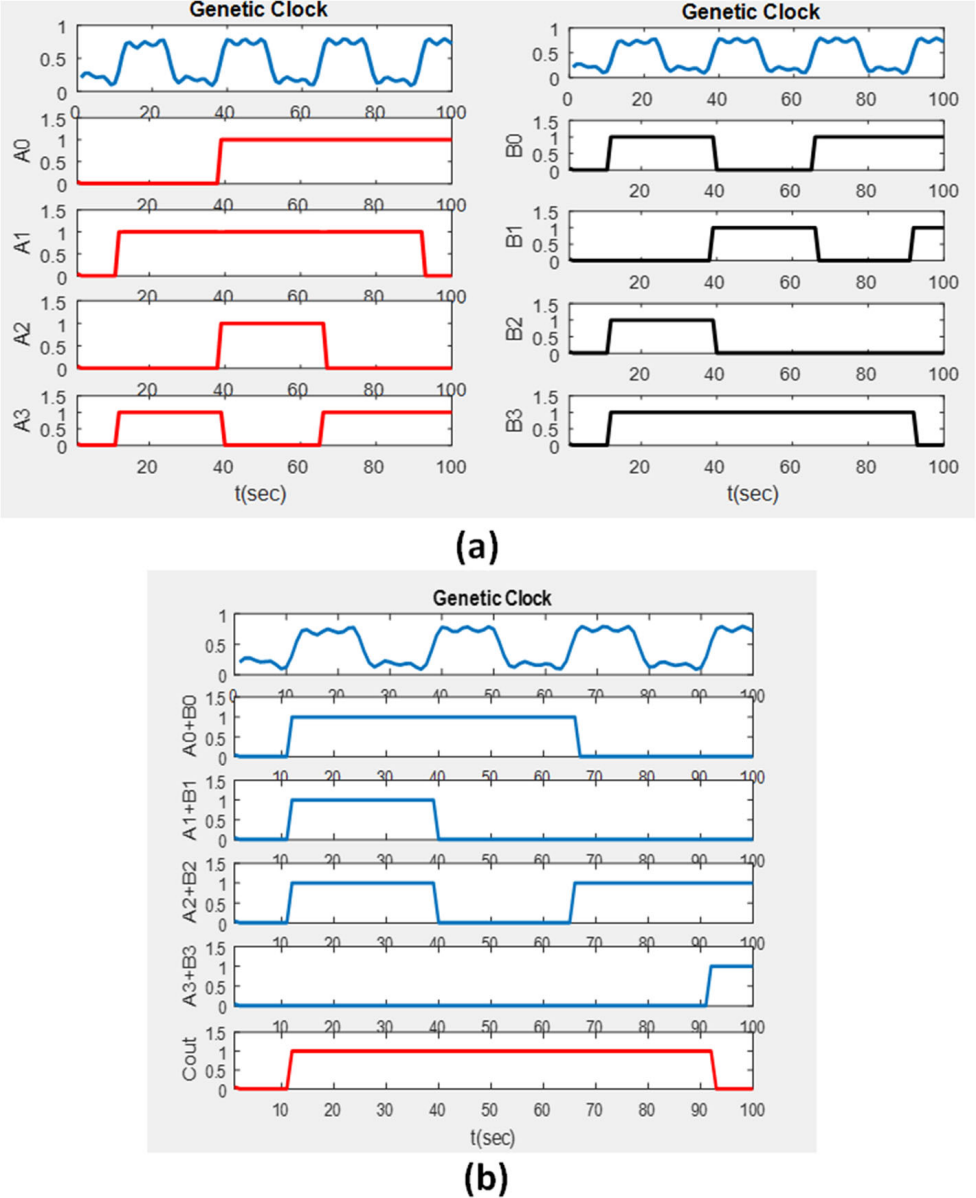
**a** Two genetic register output responses. **b** Bio-accumulator output responses. **a** Changes of concentration in the two genetic registers are data to be added together. **b** The bio-accumulator produces the result after performing binary addition

A full 4-bit addition requires five clocks to fulfill the entire work. When the instruction is triggered, all data will be placed in the register first. After the instruction being executed, the resulting data will be left in the accumulator. However, the data still needs to be stored back to the memory after execution. Figure 17a shows the bit states of the two temp registers. Figure 17b shows the resulting states of the 4-bit accumulator with a carry after performing addition.

#### Decoding of instruction

**Fig. 18:**
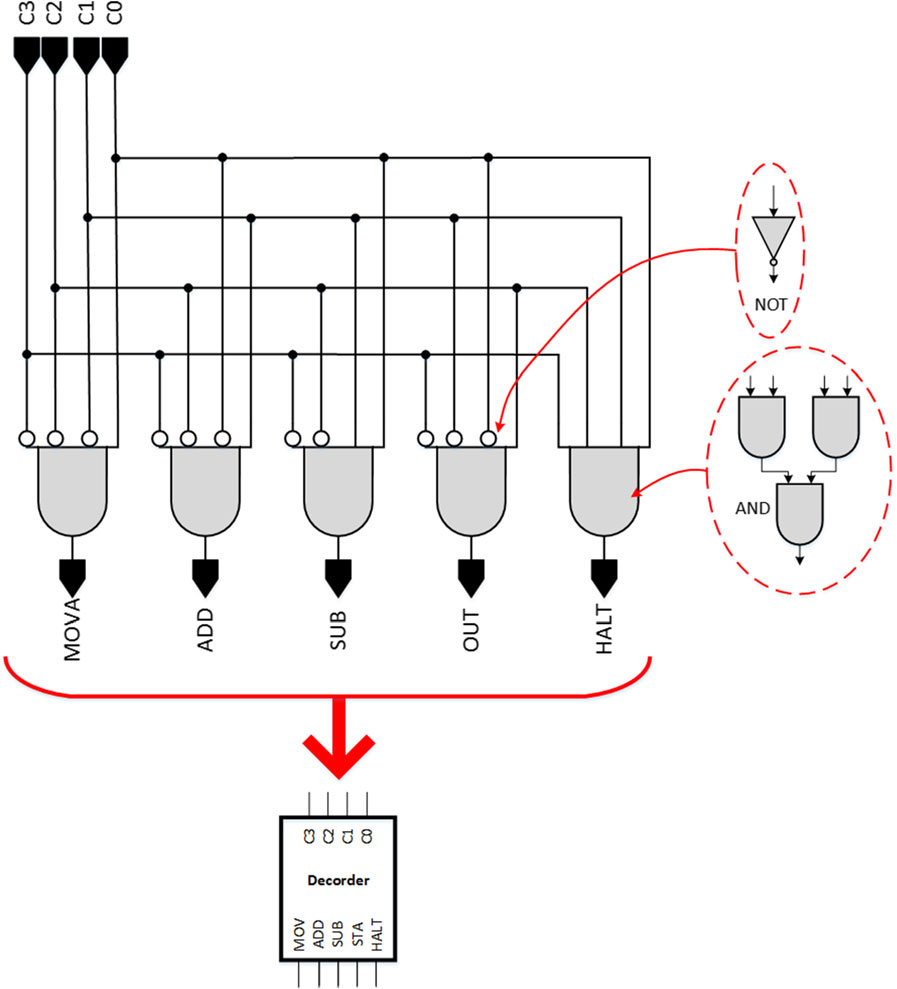
Instruction decoder. The decoder determines the instruction sets to be executed by the bio-CPU

To fulfil a specific instruction, the CU needs to determine the steps required to complete the instruction generated by the instruction decoder which reads instruction from memory. The instruction decoder takes data stored in the instruction register and decodes it. The circuit of the instruction decoder for five fundamental logic/arithmetic functions is illustrated in Fig. 18.

**Fig. 19:**
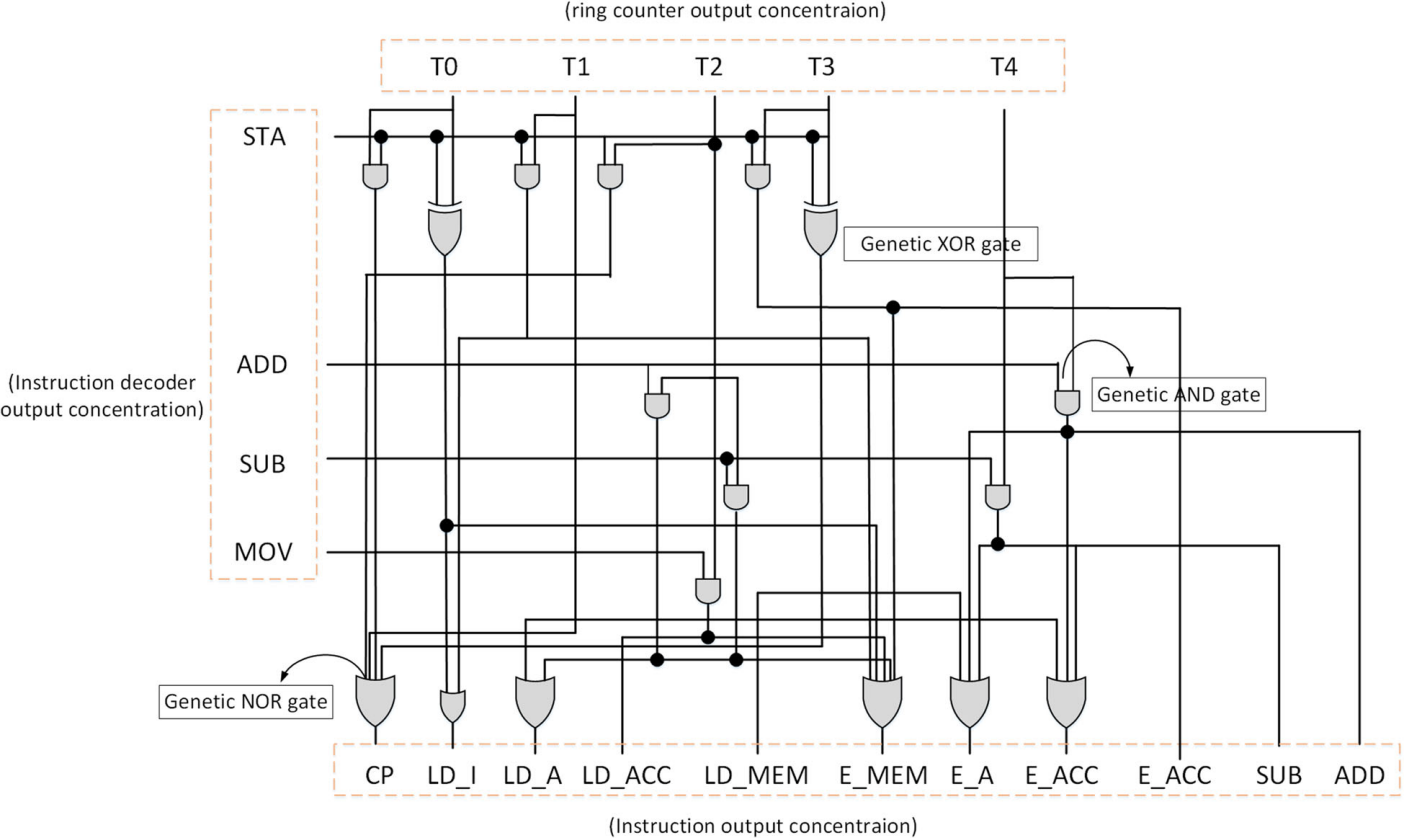
Hardware of the bio-CU. This figures displays hardware structure of the bio-CU. The logic gates in this figure are constructed by a variety of fundamental genetic logic gates. The working sequence is controlled by the bio-ring counter accompanied with the opcode

The CU system in the digital computer is established by the output of instruction decoder collocated with a ring counter and several logic gates, see Fig. 19. An instruction enters the CU system while the instruction decoder decodes the instruction. Once the instruction decoder decodes the instruction and sends a corresponding output, the CU system carries out the required steps with the ring counter to enable the selected unit. The SUB is completed in five pulses by the ring counter. From T0 to T4, the CU enables the registers inside the CU system, see Table 1 for the working sequences to conduct the arithmetic subtraction (SUB).

**Table 1:** Sequence for realizing an arithmetic subtraction (SUB)

SUB A,#n	CP	LD_I	LD_A	LD_ACC	LD_MEM	E_A	E_MEM	E_ACC	SUB	ADD	Instruction
T0	0	1	0	0	0	0	1	0	0	0	IR ← MEM
T1	1	0	0	0	0	0	0	0	0	0	PC ← PC + 1
T2	0	0	1	0	0	0	1	0	0	0	A ← MEM
T3	1	0	0	0	0	0	0	0	0	0	PC ← PC + 1
T4	0	0	0	0	0	1	0	1	1	0	ACC ← A-ACC

Table 2 shows the execution steps of the CU. Using SUB as the example. First of all, at the time instant T0, the instruction to be executed is loaded from memory to the instruction register (IR), then the program counter keeps the address of the instruction being executed at T1, and the instruction loads data to the register A from memory at T2. At T3, the program counter holds the memory address of the next instruction-the subtraction operation from the register A and accumulator. Finally, at T4, the CU executes the instruction SUB and keeps data in the accumulator. The steps for accomplishing an arithmetic subtraction are accomplished under the command directed by the CU which decomposes instruction to several data movements between memory and ALU.

**Table 2:** Operational sequence of the demonstrative instructions

Mnemonic	Effect	T0	T1	T2	T3	T4
MOVA	ACC ← Memory	IR ← Memory	PC ← PC + 1	ACC ← Memory	PC ← PC + 1	
ADD A	ACC ← ACC + A	IR ← Memory	PC ← PC + 1	A ← Memory	PC ← PC + 1	ACC ← (A + ACC)
SUB A	ACC ← A-ACC	IR ← Memory	PC ← PC + 1	A ← Memory	PC ← PC + 1	ACC ← (A-ACC)
OUT A	Memory←ACC	PC ← PC + 1	IR ← Memory	PC ← PC + 1	Memory←ACC	
HLT	STOP					

## Simulation result

**Fig. 20:**
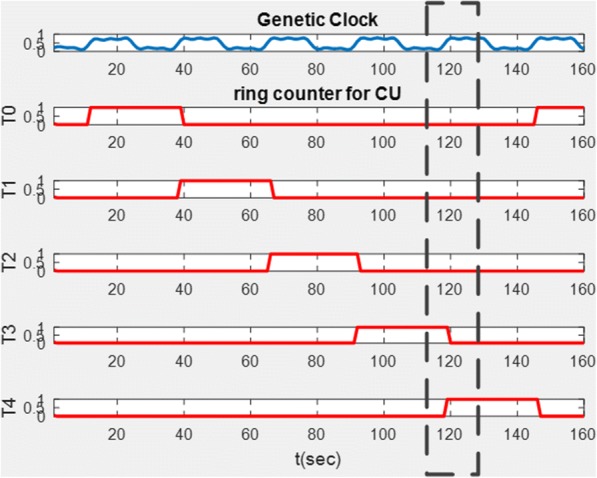
Q4 is triggered at *t* = 120. The figure demonstrates subtraction executed by the bio-CPU where the bio-ALU is triggered when T4 goes from low to high at *t* = 120

**Fig. 21:**
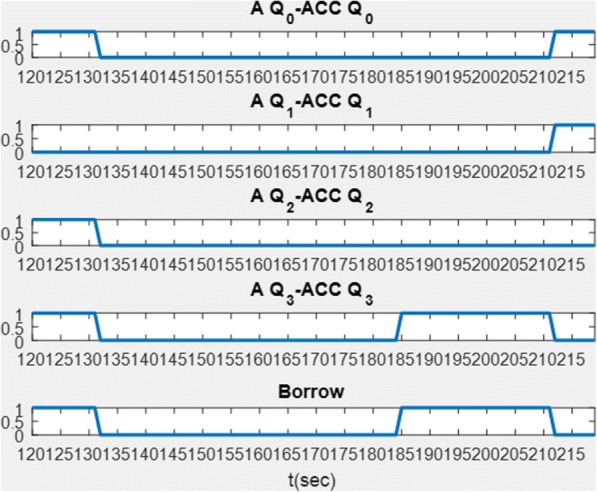
Result of subtraction. The bio-ALU executes a 4-bit subtraction

We demonstrate partial function of the instruction cycle which includes acquiring instruction from the instruction decoder and triggers ALU at the proper timing. Using the instruction SUB as an example, once the CU receives the instruction SUB from the instruction decoder, the individual steps to ensemble the instruction are activated sequentially. Fulfilment of a SUB instruction consists of five steps as illustrated in Fig. 20. The 5-stage ring counter repeats every five clock pulses and the ALU is triggered at T4, see Fig. 20. The ALU is triggered when T4 goes from low to high at *t =* 120, see Fig. 21. The data is stored back to the ACC once ALU fulfils the operation.

In terms of biological circuits, the bio-CU and bio-ALU are created under synthetic biology. All of the circuits are composed of several genetic logic gates which use protein concentration as the signal input or output, the output reaction resembles individual behaviour of a variety of logic gates. The clock signal can be generated by a genetic clock generator which can be synthesized from an oscillator with a toggle switch. While responses of all functional modules presented here are not perfect as those of their counterparts in the silicon computer, the preliminary simulation study reveals the possible reactions in terms of the genetic circuits.

## Discussion

Synthetic biological circuit design is commonly inspired by the natural biological circuits using transcription factors. There are already biological circuits revealed in the literature using the transcription factor for construction [7, 27–29], some of the circuits use recombinases as the pathways [30, 31]. In this primitive research task, the biological circuits are realized from the viewpoint of mathematics and engineering. There were biological circuits with specific purposes developed and experimentally realized [1, 11, 12, 32–36]. This research task moves forward one step by building up a class of biological circuits which possess sophisticated functions including bio-sequential circuits and bio-combinational circuits. Virtually all circuits in practical digital devices are a mixture of combinational and sequential logic which forms as a basis for constituting a functional biocomputer.

However, the biological circuits being totally replaced by biological molecules still have lots of issues to be solved in nature. Fortunately, there are some impressive progresses unveiled recently. For example, setting up measurement devices to show fluorescence concentrations of a series of repressor or activating genes with different promoter-RBS components and TF by fluorescence measurement has been developed [37]. Synthetic biologists have also created software (such as the Web-based Cello [38]) that automates the design of DNA circuits for living cells. The research team led by Prof. Voigt has developed user interfaces for Cello that would allow biologists to develop a single program and be returned different DNA sequences for targeted organisms. The library encompasses rich information of system parameters related to the mathematical model describing for the behaviors of a class of genetic logic gates. Therefore, one would be easier to choose suitable promoter RBS components to realize various biological circuits and construct them based on the proposed topology.

## Conclusions

**Fig. 22:**
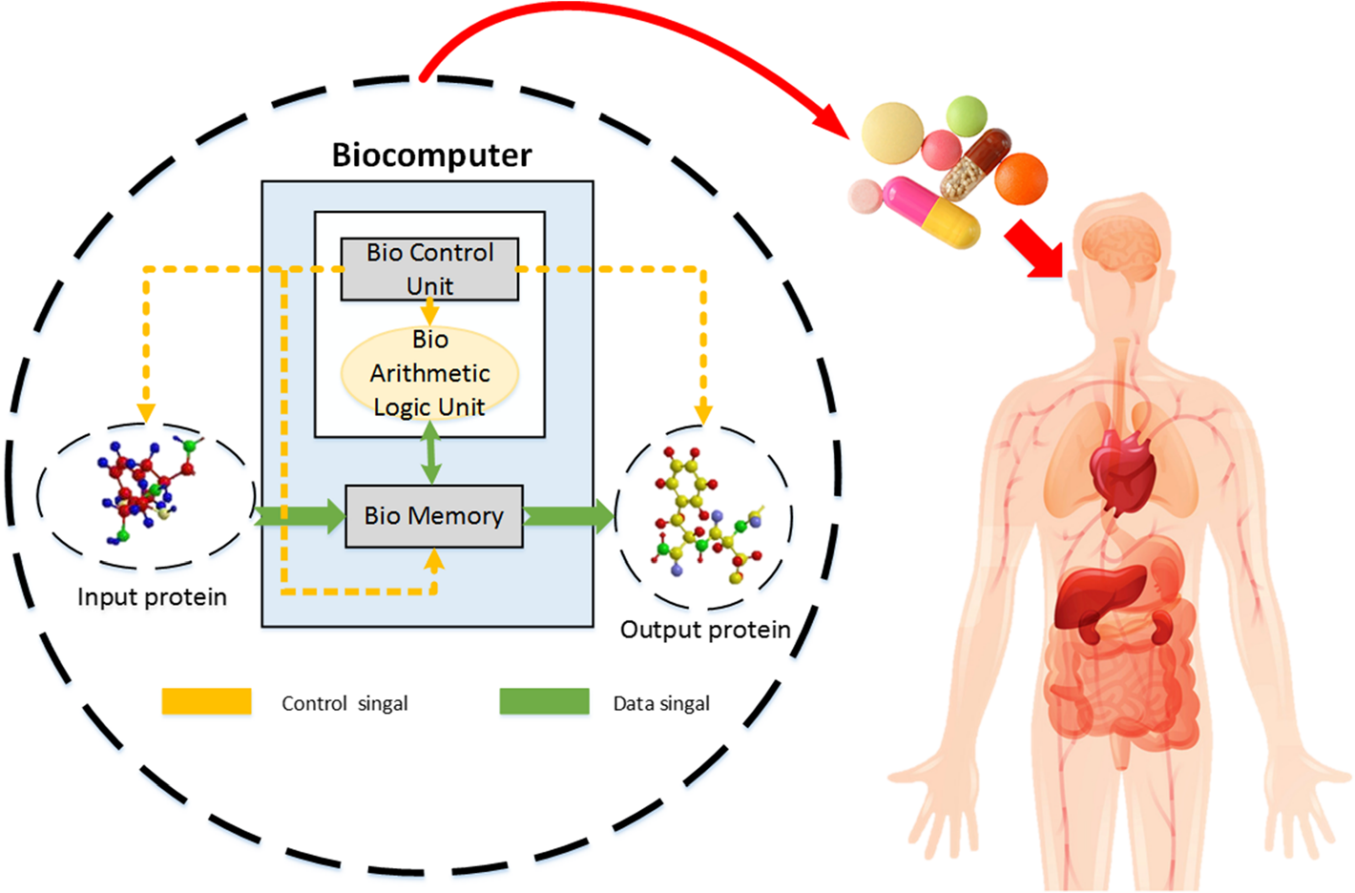
The schematic diagram of the ideal biocomputer. It represents the potential application to be realized in the biocomputer for curing genetic disease with gene therapy

Nowadays, science and technology evolve extremely fast than the past decades, the major reason should attribute the success to the invention of the computer. It can make many tedious computational works be processed by itself efficiently and manipulate a huge amount of data at one time. This paper presents an innovative idea that proposes a novel bio-CU structure based on the previously developed genetic circuits. The structure is basically referred to the fundamental silicon CPU. It is believed that if the biocomputer becomes mature in the near future, there will be increasing applications that may revolutionize breakthrough in other fields. As for applications, the DNA computer is reaching to a fair level that could be used for detection of genetic disease with gene therapy. For example, the DNA computer can measure the concentration of a specific antibody and it is possible to determine whether a patients is suffering from a particular disease. A DNA computer can determine whether a specific drug is needed based on the presence of one or more antibodies [39, 40]. Taking a biocomputer into the body from a pill is like to implant a subminiature doctor into patient’s body. Because of the function of bio-memory, it could store the information of diseases [41]. Through the transcription factors to regulate genetic sequences and produce the specific protein is equivalent to the process that one inputs the instructions to dictate the computer to execute a specific function. Figure 22 demonstrates the schematic diagram of the use of a biocomputer for the diagnosis purpose.

The idea presented here is primitive which is not rigorous enough. Actually, the idea was originated from the silicon computer. However, there were significant modifications which have been conducted in our research such as the interface handling between two modules and signal shaping of the output of gene circuits as their output responses are not fast enough. This causes the signal’s status change ambiguously leading to unavoidable delay during signal transition in the sequential circuit. Of course, there are many issues to be addressed before a full functioned biocomputer is ultimately realized.

## Abbreviations

ACC: Accumulator
ADD: Addition
ALU: Arithmetic Logic Unit
Biocomputer: Biological computer
Bio-memory: Biological memory
Bio-register: Biological register
Bio-ring counter: Biological ring counter
Bio-temporary register: Biological temporary register
CPU: Central Processing Unit
CU: Control Unit
D-FF: D Flip-flop
E_A: Enable Register A
E_ACC: Enable Accumulator
E_MEM: Enable Memory
HLT: Halt
IR: Instruction Register
LD_A: Load Register A
LD_ACC: Load Accumulator
LD_I: Load Instruction Register
MOV: Move
mRNA: messenger RNA
PC: Program Counter
PIPO: Parallel Input and Parallel Output
RNAp: RNA polymerase
SUB: Subtraction
TF: Transcription Factor
